# Nature Scenes Counter Mental Fatigue-Induced Performance Decrements in Soccer Decision-Making

**DOI:** 10.3389/fpsyg.2022.877844

**Published:** 2022-04-29

**Authors:** He Sun, Kim Geok Soh, Xiaowei Xu

**Affiliations:** ^1^Department of Sport Studies, Faculty of Education Studies, Universiti Putra Malaysia, Selangor, Malaysia; ^2^Sports Education Centre, Zhejiang Shuren University, Hangzhou, China

**Keywords:** nature exposure, mental fatigue, decision-making, soccer, performance

## Abstract

**Background:**

It has been well investigated that nature exposure intervention can restore directed attention and improve subsequent cognitive performance. The impairment of decision-making skills in mentally fatigued soccer players was attributed to the inability of attention allocation. However, nature exposure as the potential intervention to counter mental fatigue and improve the subsequent decision-making skill in soccer players has never been investigated.

**Objects:**

This study aimed to evaluate the effects of nature exposure intervention on decision-making skills among mentally fatigued university soccer players. Moreover, different durations of nature exposure were also evaluated.

**Methods:**

A random control between-subject design was adopted. Players were randomly assigned into six groups with three different durations of the experimental group compared with the corresponding control group (4.17 min: Exp 1 vs. Con 1; 8.33 min: Exp 2 vs. Con 2; and 12.50 min: Exp 3 vs. Con 3). All players were first mentally fatigued by performing a 45-min Stroop task; then, they viewed virtual photos of natural or urban scenes; and finally, they performed a soccer decision-making task.

**Results:**

The subjective ratings of mental fatigue were significantly higher following the Stroop task. Only Exp 3 (12.50 min viewing natural scenes) significantly improved decision-making reaction time compared with Con 3 (*p* = 0.09). Moreover, the accuracy slightly increased in Exp 3 after the intervention.

**Conclusion:**

In line with attention restoration theory, nature exposure significantly improved decision-making skills in mentally fatigue university players. However, the duration must be 12.50 min for each stimulus to stay longer to attract involuntary attention.

## Introduction

Human have been evolving over the past 200,000 years, and they have grown in response to the natural environments (Berto et al., 2018). This response is cemented in our evolutionary history due to biophilia, which is why people become aware of the positive benefits associated with exposure to the natural environment, such as attention restoration (Berto et al., 2018) and mental health (Grinde and Patil, 2009; Beyer et al., 2014; van den Bosch et al., 2015).

Notably, James identified two forms of attention, each distinguished by the level of effort required to use them. One type referred to as voluntary attention (directed attention) is effortful and tiring. In contrast, involuntary attention is unpromoted and effortless. Especially, it allows the attentional system to have a rest and recovery (1982 as cited in Kaplan and Berman, 2010). Based on this, attention restoration theory (ART) was proposed to explain the directed attention restoring procedure (Kaplan and Berman, 2010). Furthermore, Berto et al. (2010) argued that ART offers an approach to understanding fatigue or depletion and suggests restoring this resource. The effort of attention can be avoided if individuals can surround themselves with enough involuntarily interesting things (Berto et al., 2010; Kaplan and Berman, 2010). These exciting things mainly exist in highly engaging environments, which is nature (Berto et al., 2010). Conceptually, nature exposure refers to a “direct physical or sensory contact with the natural environment” (e.g., sitting in a natural environment and gardening) (Kamitsis and Francis, 2013). The positive effect of nature exposure depends on the degree of immersion, which means the effects of nature exposure may be more robust when people are more fully immersed in these environments (Berto et al., 2018). It has been well investigated that the exposure can be done in a virtual environment (Berto, 2005; Beute and de Kort, 2014; Pilotti et al., 2015), and even, the duration of applying nature exposure intervention in a virtual environment is shorter than that of the natural environment (Berman et al., 2008; Stevenson et al., 2018), which is highly eye-catching in this pandemic.

Mental fatigue is a fatigue condition, generally induced by increased demand for cognitive actions, which is usually recognized as a complex phenomenon of psychophysiology (Hancock and Desmond, 2001). Characteristically found among the open-skill sports (e.g., soccer), which requires players to react in an unpredictable and dynamically changing environment (Holfelder et al., 2020) with extremely challenging cognitive demands (Heppe et al., 2016; Holfelder et al., 2020). In particular, all teammates' and opponents' trajectory paths, as well as the ball, are usually dynamic and unpredictable; objects frequently blocking from view, as well as various occlusions and segmentation, may interrupt players' original directions at any time. Thus, players must maintain alertness throughout the game that raises the chances to be mentally fatigued. Especially, soccer players may be more susceptible to mental fatigue due to the prolonged duration of exposure to cognitive activities (Smith et al., 2018; Sun et al., 2021). Numerous studies have shown that there are negative effects of mental fatigue on soccer performance (Fortes et al., 2019, 2020; Gantois et al., 2019; Soylu et al., 2022). However, the intervention is still misty.

Notably, the impairment in skilled performance (e.g., decision-making skill) was theoretically attributed to a reduction in executive functions (e.g., directed attention) due to mental fatigue tasks activating the anterior cingulate cortex, likely leading to elevated adenosine and correspondingly decreased dopamine (Smith et al., 2018; McMorris, 2020; Sun et al., 2021). In soccer, decision-making highly relies on the human brain's ability to perceive relevant information from a complex environment while blocking out irrelevant distractions (Baker et al., 2003; Gantois et al., 2019). This process is controlled by directed attention (Kastner et al., 1999; Murray and Wojciulik, 2004). However, it was impaired by mental fatigue.

Moreover, the effects of mental fatigue could also be explained by the strength model of self-regulation (Baumeister and Heatherton, 1996). According to the model, all self-regulation processes draw on the same limited brain resource, and therefore, the subsequent task performance that requires effortful attention was impaired (Baumeister et al., 2007 for a review). Consistently, Furley et al. (2013) demonstrated that insufficient self-regulatory resources impaired sports decision-making since the cognitive functioning of attention was decreased. Notably, directed attention was recognized to be a cross-domain resource in self-regulation and executive function (Kaplan and Berman, 2010).

Therefore, when nature exposure restores directed attention in mentally fatigued players, they may better concentrate on retrieving information, block irrelevant stimuli (e.g., cluster or/and competitive state anxiety), and improve their decision-making as mental fatigue is prevented or minimized.

However, the duration of nature exposure is diverse in the previous evidence (Berto, 2005; Chow and Lau, 2015; Pilotti et al., 2015). Cognitive tasks of prior mental exertion partially determine the efficiency of interventions. To investigate the question, Beute and de Kort (2014) carried out two studies. In study one, they used a 3-min virtual nature exposure to counter a typing task and improve the subsequent performance significantly. Nevertheless, the same intervention lost efficiency when adding a 4-min Stroop task to the prior mental exertion. Therefore, it is essential to evaluate different durations of nature exposure intervention and determine the optimal choice. This study sets the first investigation by Berto (2005) in this field as the standard. Considering that the prior cognitive task (sustained attention to response test, SART) is 5 min in Berto (2005), which is much shorter than the 45-min Stroop task recruited in this study, subjects may be less mentally fatiguing. Thus, this study prolonged viewing each photo from 15 to 20 s (viewing 25 photos: 8.33 min) and 30 s (viewing 25 photos: 12.50 min). Moreover, since Berto (2005) mentioned that SART is very mentally fatiguing, this study also shortened viewing each photo from 15 to 10 s (viewing 25 photos: 4.17 min).

Moreover, previous research mainly focused on the level of elite players (e.g., Gantois et al., 2019; Fortes et al., 2020), for they experience extreme pressure, and coupled with high training and competition demands, an enormous potential for mental fatigue exists. Nevertheless, compared with elite players, university players face pressure from routine training and extra-occupational cognitive load, such as school work (Seibert et al., 2016). Consequently, mental fatigue may be easily bound to university players and impair their soccer decision-making. Therefore, this study evaluates the effect of nature exposure intervention on mentally fatigued university soccer players. Moreover, three different durations (i.e., 4.17, 8.33, and 12.50 min) are examined to find the optimal choice.

## Methods

### Participants

The sample size was calculated using G^*^Power 3.1 software. The effect size calculation was based on a previous study's declarative knowledge (Wang et al., 2016), which was done in China. Additionally, an alpha level of 0.05 and a power of 0.80 were established. Eventually, university male players (age: 20.73 ± 2.00 years; duration of training: 5.14 ± 1.31 years) were recruited voluntarily, who came from two local universities' soccer teams competing at the province level (Henan, China).

Written consent was provided for the players to sign for participation in the study. Moreover, the approval letter was issued by the Universiti Putra Malaysia Ethics Committee (Project #: JKEUPM-2020-327) and conformed to the Declaration of Helsinki. The protocol was registered in ClinicalTrial.gov on 05/01/2021 with Identifier NCT04693481. The study's objectives were not disclosed to participants, but they were informed that it was “investigating soccer-specific skill after a common pre-match activity.”

Participants should fulfill the following criteria. Inclusion criteria are (i) male players aged 18–24 years, (ii) at least 3 years of training experience, and (iii) no sleeping difficulties. Exclusion criteria are (i) goalkeepers, (ii) color blindness, and (iii) experienced any psychological technique or had a psychological background.

### Development of Stimuli Material

To guarantee the efficiency of the experiment, the development of stimuli material from nature photos is crucial. Nature sceneries are usually free of human-made objects but do not entirely lack human control, such as national forests or national parks (Balling and Falk, 1982). From a broader viewpoint, Asian adults tend to regard scenes as natural when they fulfill three criteria: (i) the scenery is overshadowed by vegetation, water, and mountains; (ii) non-natural features are devoid of hidden; and (iii) the predominant contours are curvilinear or irregular (Ulrich, 1983; Han, 2003).

Another criterion should also be paid attention to, as demonstrated by some studies. Staats (2012) and Wang et al. (2016) reported the results of the experiments: nature scenes without people were more restorative than scenes depicting people. They named this character the “absence of people.” Therefore, according to the definition of nature scenes and some other manifestations of restorative environments, this study selected various nature scenes with the “absence of people.”

First, a vast collection of photos of natural landscapes was supplied by the photography department at Zhongyuan University of Technology (ZUT) in China, which many instructors shot during previous outdoor photography courses. Next, to adhere to the criteria for photos, two experts (refer to details in Table 1) were recruited to select appropriate photos. The first expert evaluated photos based on the definition of nature scenes (Balling and Falk, 1982) and the expansion toward Asian adults (Ulrich, 1983; Han, 2003). The second expert assessed pictures for photography quality to verify that they were selected appropriately and did not include any distortion of colors or forms, and eye-level horizontal views. Fifty color photographs were eventually selected, representing different nature scenes, such as lakes, rivers, seas, hills, mountains, forests, and grassland.

**Table 1:** List of two experts for stimuli material.

**Ref**	**Designation**	**Area of specialization**	**Institution**	**Position**
1	Professor	Environmental Design	ZUT	Lecturer
2	Assoc. Professor	Photographic Aesthetics	ZUT	Lecturer

*ZUT, Zhongyuan University of Technology*.

The following development process followed the study of Berto (2005). At ZUT, 40 male students (mean age = 20.90 years, SD = 2.00) aided in developing the experiment's stimulus material. Each scene was split into five groups of 10 randomly selected photographs; eight male students evaluated each group of 10 photographs. The restorative value of each scene was evaluated by each participant using the perception restorativeness scale. Individuals and small groups (maximum four students each time) were examined in the photography department's laboratory. The researcher started by introducing two practice slides to acclimate individuals to the task. Following that, five slides were projected and evaluated. Following that, a short distraction exercise (counting backward by seven from 100 to 0) was employed to prevent participants from becoming familiarized with the activity. Following that, the last five slides were then projected and evaluated. Finally, a mean across perception restorativeness scale was used to select the stimuli photos. Photos with scores in the top 25 (mean = 9.46 to 7.36) were chosen.

In addition, due to the COVID-19 situation, the researcher could not get photos from an urban environment for the control groups. Therefore, the researcher employed the urban photos from Berto (2005) to compare with natural stimuli in this study with approval.

### Measures

The study instruments mainly had three purposes of measurement, given as follows: (i) dependent variable measurement (decision-making index); (ii) mental fatigue measurement: Stroop task and Visual Analog Scale (VAS); and (iii) control variables measurement (e.g., Immersive Tendency Questionnaire [ITQ], Brief Trait Self-Control Scale, Perception of Restorativeness Scale, and Borg 10). The details of these instruments are explained in the following section.

#### Decision-Making Index

The decision-making was assessed by the TacticUP platform on the website (https://www.tacticup.com.br/en/), which is based on real-match situations. The subjects were assessed concerning their time to decide in different situations within the game and their accuracy.

The recent study by Machado and da Costa (2020) proved adequate validity and reliability in measuring soccer decision-making. The authenticity of the material was verified by a panel of nine experts from four different nations. Construct validity was established by comparing players with varying degrees of competence. When the final scores of the groups were compared, statistical differences (*p* < 0.05) were observed, with the expert groups displaying higher values than the non-expert groups. Face validity was obtained from the examination of the acceptability and suitability test by players. Reliability has been determined through the test-retest method for each video sequence, where Cohen's kappa values range from 0.62 to 1.00.

#### Stroop Task

Mental fatigue was induced using the 45-min computer-version Stroop task created by the software “E-Prime.” Several studies have shown that the computer-version Stroop task can induce mental fatigue significantly in soccer players (Smith et al., 2015, 2016b; Moreira et al., 2018; Gantois et al., 2019; Trecroci et al., 2020).

#### Visual Analog Scale

The VAS was first developed by two employees of Scott Paper Company and was used to rate subordinate workers. Ahearn (1997) successfully demonstrated that VAS is a valuable tool to measure mood. Tested on sports players, it effectively measures mental fatigue (e.g., Badin et al., 2016; Smith et al., 2016a,b; Russell et al., 2019). In order to rate the mental fatigue levels, subjects moved a single vertical line to reflect “mental fatigue,” a 100-mm horizontal line. VAS has two anchors: “0” is “none at all,” and “100” is “maximal” fatigue. The VAS was also used to measure motivation toward an upcoming task.

#### Immersive Tendencies Questionnaire (ITQ)

The ITQ was created by Witmer and Singer (1998) and used to directly identify people who would be more likely to immerse themselves in virtual environments. Everyday activities were used to measure an individual's involvement in the 18 items. Some items are related directly to IT, whereas others measure people's present alertness or fitness, while others focus on people's capacity to redirect their attention. Individuals rate their experiences on a 7-point Likert scale which ranges from 1 (not at all) to 7 (completely); the Cronbach's alpha value is 0.81 (Witmer and Singer, 1998).

#### Borg 10

To utilize the Borg 10, participants responded to the question: “How was your training?” They were required to evaluate the session's intensity on a scale of 0 to 10 (0 being “rest” and 10 being “maximum effort”).

### Design and Procedure

**Figure 1:**
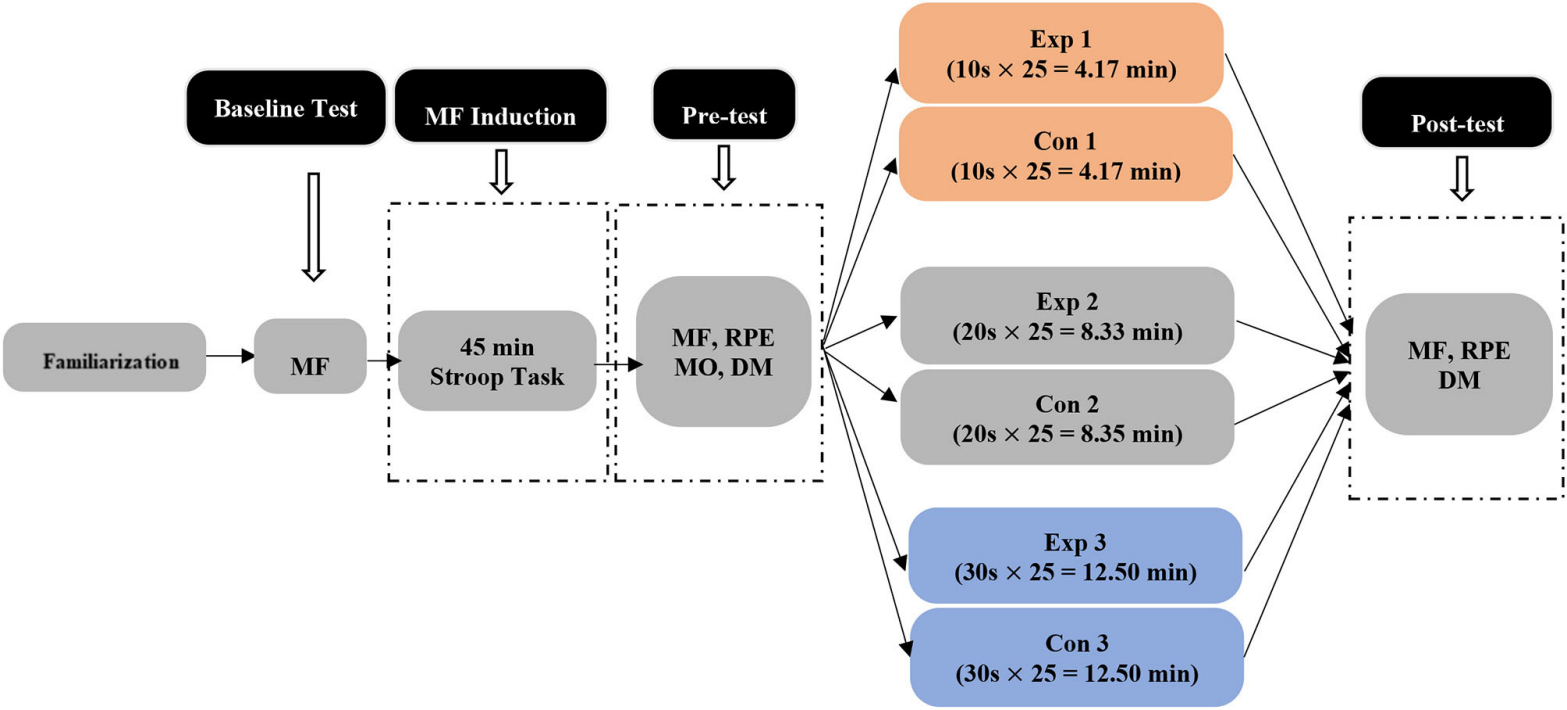
Experimental design. MF, Mental fatigue; MO, motivation; RPE, rating perception of effort; Exp 1, experimental group 1; Con 1, control group 1; Exp 2, experimental group 2; Con 2, control group 2; Exp 3, experimental group 3; Con 3, control group 3.

This was a controlled and randomized experimental between-subjects study with two visits (familiarization and testing session) for the experimental and control groups. Male soccer players were randomly assigned into six groups (Exp 1 vs. Con 1; Exp 2 vs. Con 2; and Exp 3 vs. Con 3; refer to Figure 1) by Research Randomizer (https://www.randomizer.org/).

Before the experiment, all players were pre-screened for trait-immersive tendencies by ITQ. It was used to ensure that traits in each group were similar. After recruitment, players completed two sessions (familiarization and testing session). The familiarization session included a description of the instruments. Additionally, participants were advised to sleep for 8 h, abstain from alcohol, avoid strenuous exercise within 24 h after each intervention session, and abstain from coffee/caffeine and mentally taxing activities on the day of testing.

In the testing session, a 45-min computer-version Stroop task was performed, four words (i.e., red, blue, green, and yellow) were shown one at a time, and participants were instructed to disregard the word's meaning in favor of identifying the word's color. However, if the word's ink color was red, the correct answer was determined by its meaning rather than its color. Players were instructed to hit the answer box button that matched the color of the word shown on the computer screen. Each word was shown on the screen for 1,000 ms in a 100-point typeface, followed by a 1,500 ms blank screen until the following word was displayed. Thus, for every 2,500 ms, a new word was shown, totalling 1,080 stimuli throughout the 45-min therapy. A beep from the computer speakers indicated any missing or erroneous answers (>1,700 ms).

In the TacticUP platform, players completed an answer sheet in response to the cue question “What should the observed player do?” based on a prior real clip of a soccer match (for details of the process, refer to Machado and da Costa, 2020).

In addition, all the subjects began the testing session at 10.30 a.m., and there was no class before testing. Additionally, coaches established a competitive atmosphere where participants were pushed to perform the Stroop task and decision-making test as accurately and quickly as possible to enhance stress.

### Intervention

After the pretest, the intervention proceeded. At a distance of 1 m, players would sit in front of a computer. The area was darkly lit and devoid of distractions to force them to focus on the screen. The instructor informed the participants that: (a) a sequence of photographs of natural settings will be shown; (b) there would be no other task, and they could look at the photos freely. The three experimental groups watched 4.17 min, 8.33 min, and 12.50 min of virtual nature photos, respectively, while control groups watched urban photos. After the intervention block, players were provided the post-test measurement.

### Statistical Analysis

The Skewness and Kurtosis tests were used to evaluate the distribution of data. The Leven test was used for homogeneity. All data are presented as mean and standard deviation. A one-way ANOVA was carried out to test the screening data of immersive tendency and social demographics in six groups to ensure homogeneity. Moreover, two-way repeated measures were used to analyse the six groups (Exp 1 vs. Con 1; Exp 2 vs. Con 2; Exp 3 vs. Con 3). Test interaction (pretest vs. post-test) for decision-making index, mental fatigue condition, and rating perception of effort (RPE) was observed. Finally, Bonferroni *post-hoc* tests were applied to detect possible statistical differences. All statistical analyses in this study were conducted using SPSS (26.0 IBM, NY, USA), with a significant level of *p* < 0.05.

## Results

Table 2 shows the mean and standard deviation of all variables, and there were no significant differences in ITQ (*p* = 0.97), which means the trait of engaging or immersing and an individual's attentiveness to demanding tasks are the same among the six groups. Moreover, the homogeneity of demographic characteristics was also shown in each group (age: *F* = 0.04, *p* = 0.99; duration of training: *F* = 0.14, *p* = 0.98).

**Table 2:** Mean scores (plus standard deviations) for variables.

**Variable**	**Experimental group**									
	**Exp 1**	**Con 1**	**Exp 2**	**Con 2**	**Exp 3**	**Con 3**	**Effects**	***F***	***P***	**η^2^**
**DMI accuracy (** ***n*** **)**										
Pretest	66.73 (4.25)	66.40 (5.41)	67.13 (6.44)	67.40 (6.13)	66.27 (6.91)	67.20 (5.29)	Group	0.31	0.91	0.02
Post-test	67.00 (5.28)	65.60 (6.75)	69.27 (6.94)	66.93 (5.61)	69.13 (4.78)^*^	66.87 (4.81)	Test	1.40	0.24	0.02
							Interaction	1.44	0.22	0.08
**DMI reaction time (s)**										
Pretest	7.37 (1.4)	7.47 (1.66)	7.28 (2.08)	7.30 (1.64)	7.24 (1.72)	7.33 (2.16)	Group	1.50	0.20	0.08
Post-test	6.62 (1.51)	7.55 (2.09)	6.33 (1.93)^*^	7.28 (1.40)	5.01 (1.46)^*^^#^	7.21 (1.65)	Test	16.50	<0.01	0.16
							Interaction	4.76	<0.01	0.22
**Mental fatigue**										
Baseline test	2.78 (1.52)^*^	27.13 (1.14)^*^	2.59 (1.17)^*^	2.42 (1.15)^*^	2.71 (1.06)^*^	2.41 (1.21)^*^	Group	0.19	0.97	0.01
Pretest	3.61 (1.25)	3.50 (1.09)	3.47 (1.04)	3.53 (1.62)	3.65 (0.87)	3.37 (1.32)	Test	55.02	<0.01	0.40
							Interaction	0.15	0.98	0.01
**RPE**										
Baseline test	1.60 (1.34)^*^	1.43 (1.07)^*^	1.40 (0.95)^*^	1.50 (1.09)^*^	1.57 (0.99)^*^	1.43 (1.07)^*^	Group	0.23	0.95	0.01
Pretest	5.13 (1.69)	5.27 (1.49)	5.47 (0.92)	5.07 (1.16)	5.13 (1.25)	5.00 (1.13)	Test	262.02	<0.01	0.76
Post-test	4.53 (1.64)	4.60 (1.55)	4.60 (1.59)	4.33 (1.50)	4.20 (1.31)	4.07 (1.75)	Interaction	0.25	0.99	0.02
ITQ	65.40 (5.14)	65.07 (4.67)	64.87 (3.82)	65.07 (5.20)	65.47 (7.03)	64.53 (5.63)	Group	0.06	0.97	
Age	20.73 (2.37)	20.53 (1.96)	20.87 (2.20)	20.73 (2.02)	20.73 (1.94)	20.80 (1.82)	Group	0.04	0.99	
DOT	5.33 (1.35)	5.00 (1.73)	5.20 (1.15)	5.13 (1.30)	5.00 (1.25)	5.20 (1.21)	Group	0.14	0.98	

* *p < 0.05 vs. corresponding variables' pretest*;

# *p < 0.05 vs. corresponding control groups*;

*DMI, decision-making index; RPE, rating perception of effort; ITQ, Immersive Tendency Questionnaire; DOT, duration of training*.

**Figure 2:**
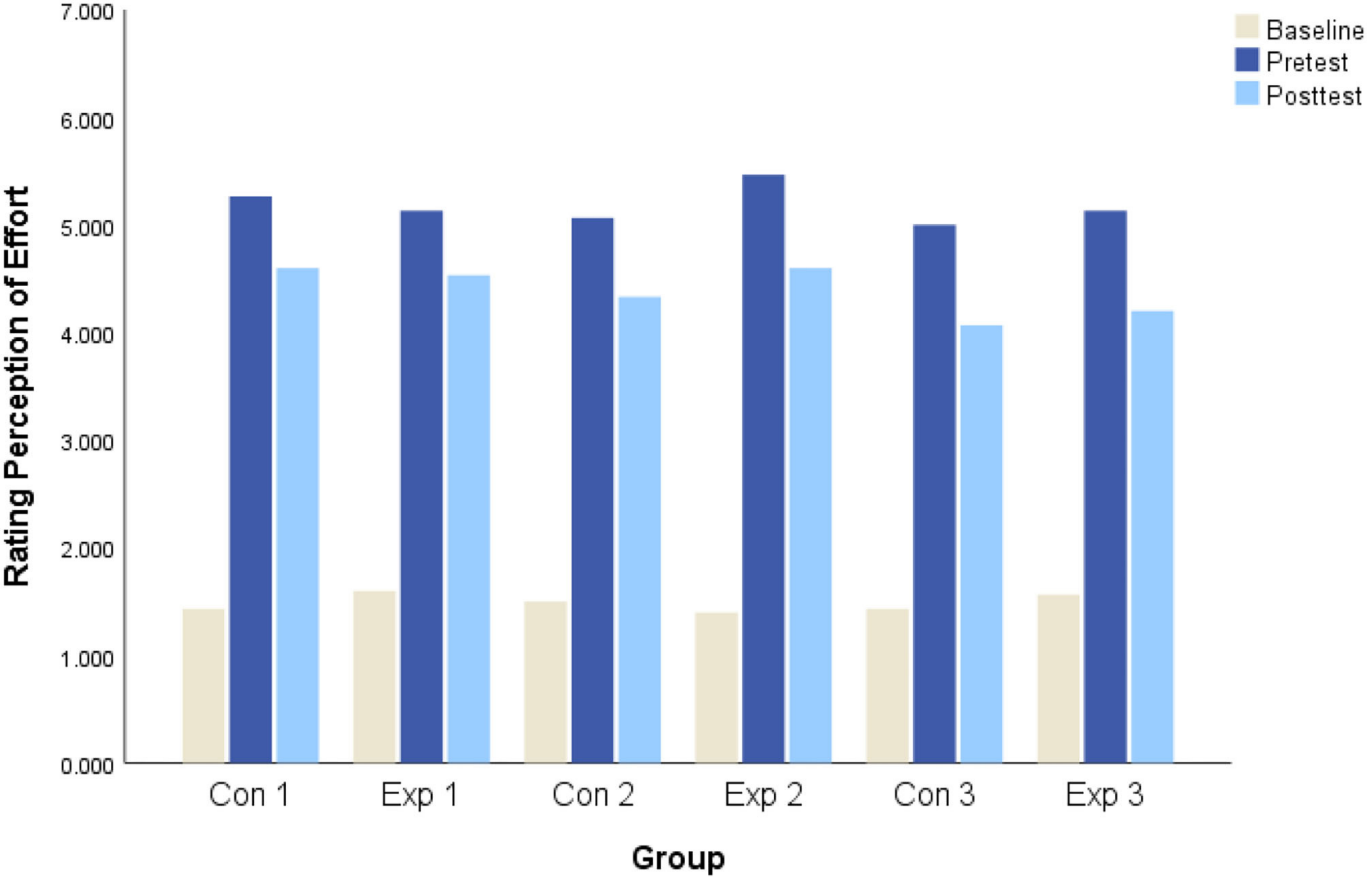
The changes in rating perception of effort in each group.

A significant within-subject test existed for mental fatigue condition (*F* = 55.02, *p* < 0.01, η^2^ = 0.40). Follow-up tests revealed higher subjective ratings in the mental fatigue existed among the six groups at post-test. Moreover, there were no significant differences in mental fatigue at pretest and post-test among the six groups. RPE was significantly increased from baseline test to pretest (*p* < 0.1) (Figure 2). Furthermore, it dropped a bit from pretest to post-test. No significant differences were shown among the six groups in each test.

Regarding the reaction of decision-making, the results (Table 2) show that the within-subjects effect (test) was statistically significant (*F* = 16.50, *p* < 0.01, η^2^ = 0.16). Moreover, the statistical significance was found in the interaction (*F* = 4.76, *p* < 0.01, η^2^ = 0.22), indicating the increase in reaction time was substantially varied over time for six groups (pretest and post-test). In addition, no statistical significance (*F* = 1.50. *p* = 0.20, η^2^ = 0.08) was found in the group impact.

According to the Bonferroni *post-hoc* test, the changes in reaction time mean scores between pretest and post-test were statistically significant (*p* < 0.05) in Exp 2 and Exp 3. Contrastingly, no significant differences were discovered in experimental group 1 and in the three control groups from pretest to post-test (*p* > 0.05). Moreover, a significant variance was found between Exp 3 and Con 3 at post-test (*p* = 0.01). There were no significant variances in the other corresponding control and experimental groups (*p* > 0.05) at pretest and post-test.

**Figure 3:**
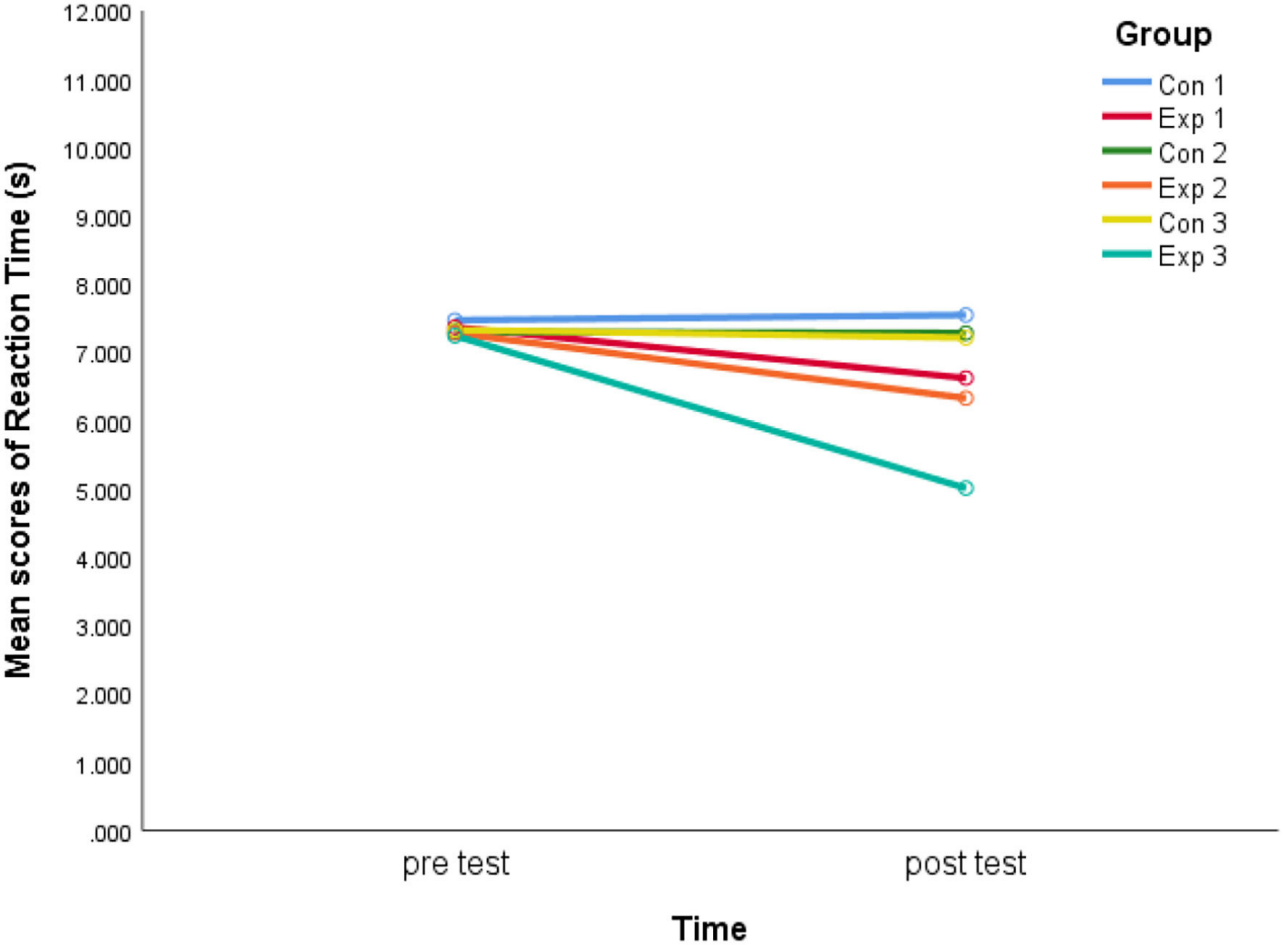
Interaction effect for reaction time.

Figure 3 illustrates how reaction time varied slightly between pretest and post-testing in the three control groups. At the same time, there was a dramatic decrease in experimental group 3 and reached the lowest level, following experimental group 2. Experimental group 1 dropped the least in all three experimental groups.

Regarding the accuracy of decision-making, the results (Table 2) for the within-subjects effect (test) were not insignificant (*F* = 1.40, *p* = 0.24, η^2^ = 0.02). Findings suggest that there was no statistical significance in the interaction (*F* = 1.44, *p* = 0.22, η^2^ = 0.08), indicating the variations in accuracy among the six groups were not statistically significant (pretest and post-test). In addition, no statistical significance (*F* = 0.31, *p* = 0.91, η^2^ = 0.02) was found in the group impact.

According to the Bonferroni *post-hoc* test, the difference in the mean accuracy of decision-making scores between pretest and post-test in experimental group 3 was statistically significant (*p* = 0.03). Contrastingly, no significant variances were discovered in experimental groups 1 and 2, and in the three control groups from pretest to post-test (*p* > 0.05). Furthermore, there were no significant fluctuations in pretest and post-test scores between the respective control and experimental groups (*p* > 0.05).

**Figure 4:**
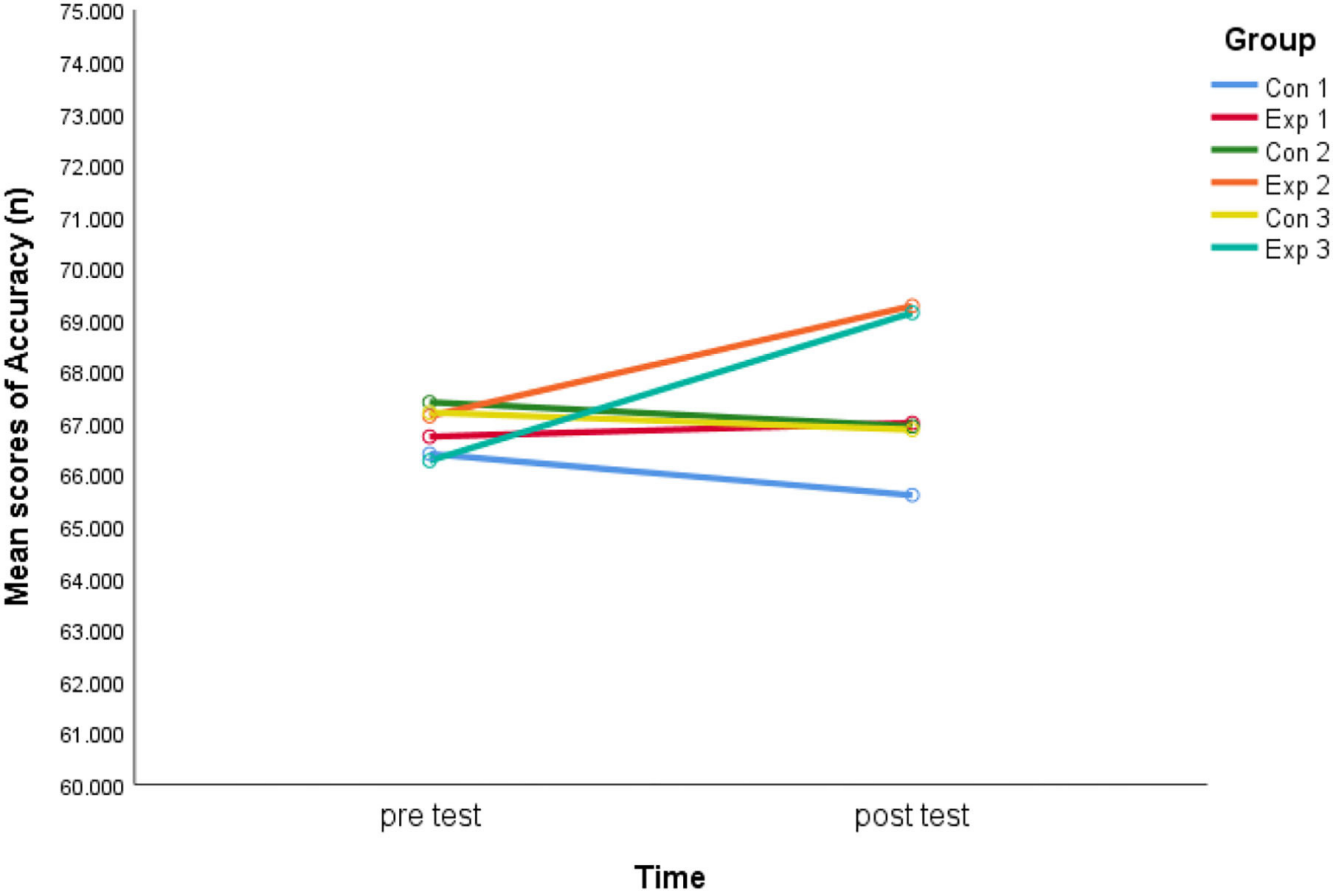
Interaction effect for accuracy.

Figure 4 shows that the accuracy decreased somewhat from pretest to post-test in the three control groups, but it increased in the three experimental groups.

**Figure 5:**
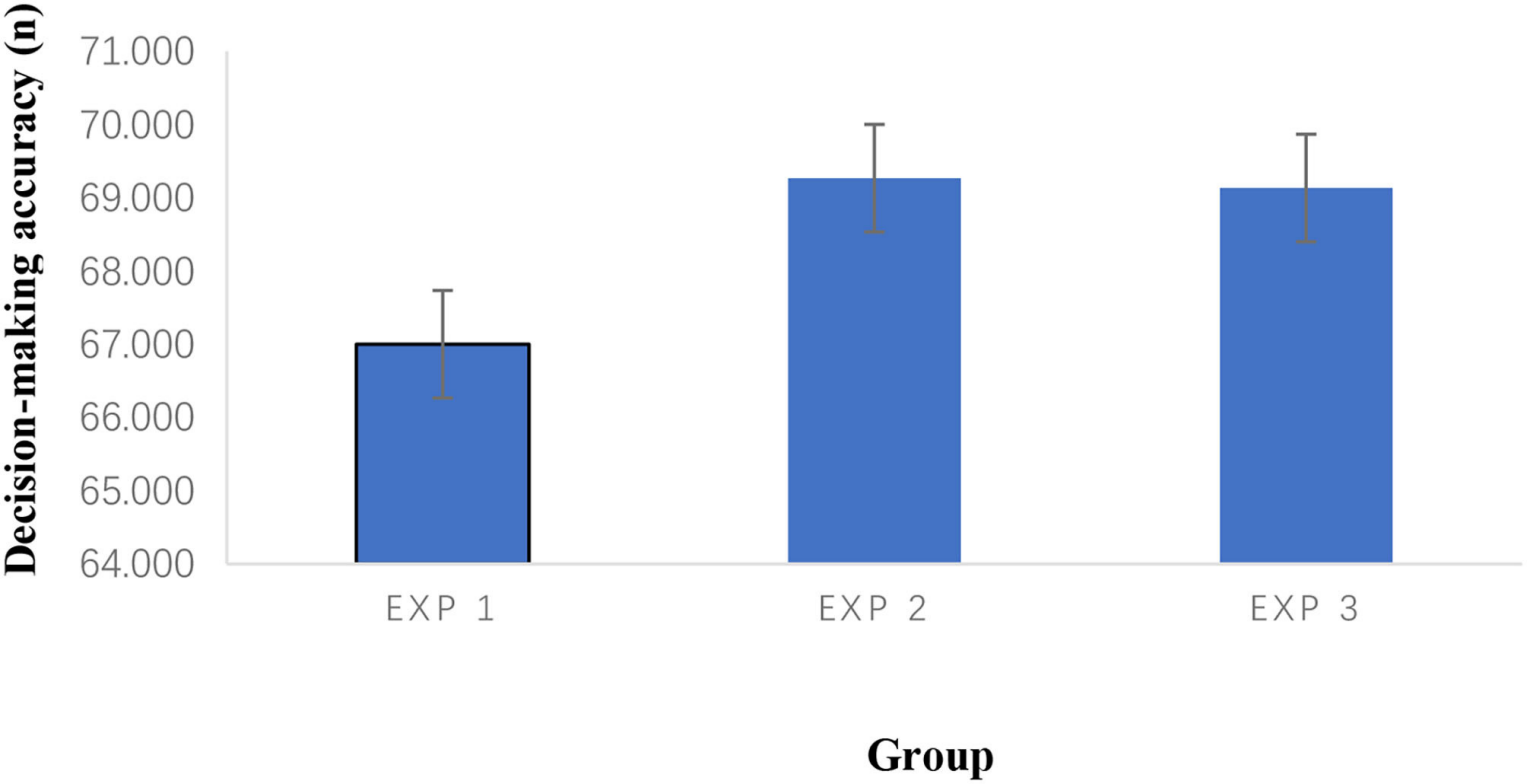
The mean of accuracy at post-test.

Figure 5 shows that there is a dramatic increase in accuracy from Exp 1 to Exp2. The accuracy almost maintained the same level between Exp 2 and Exp 3.

**Figure 6:**
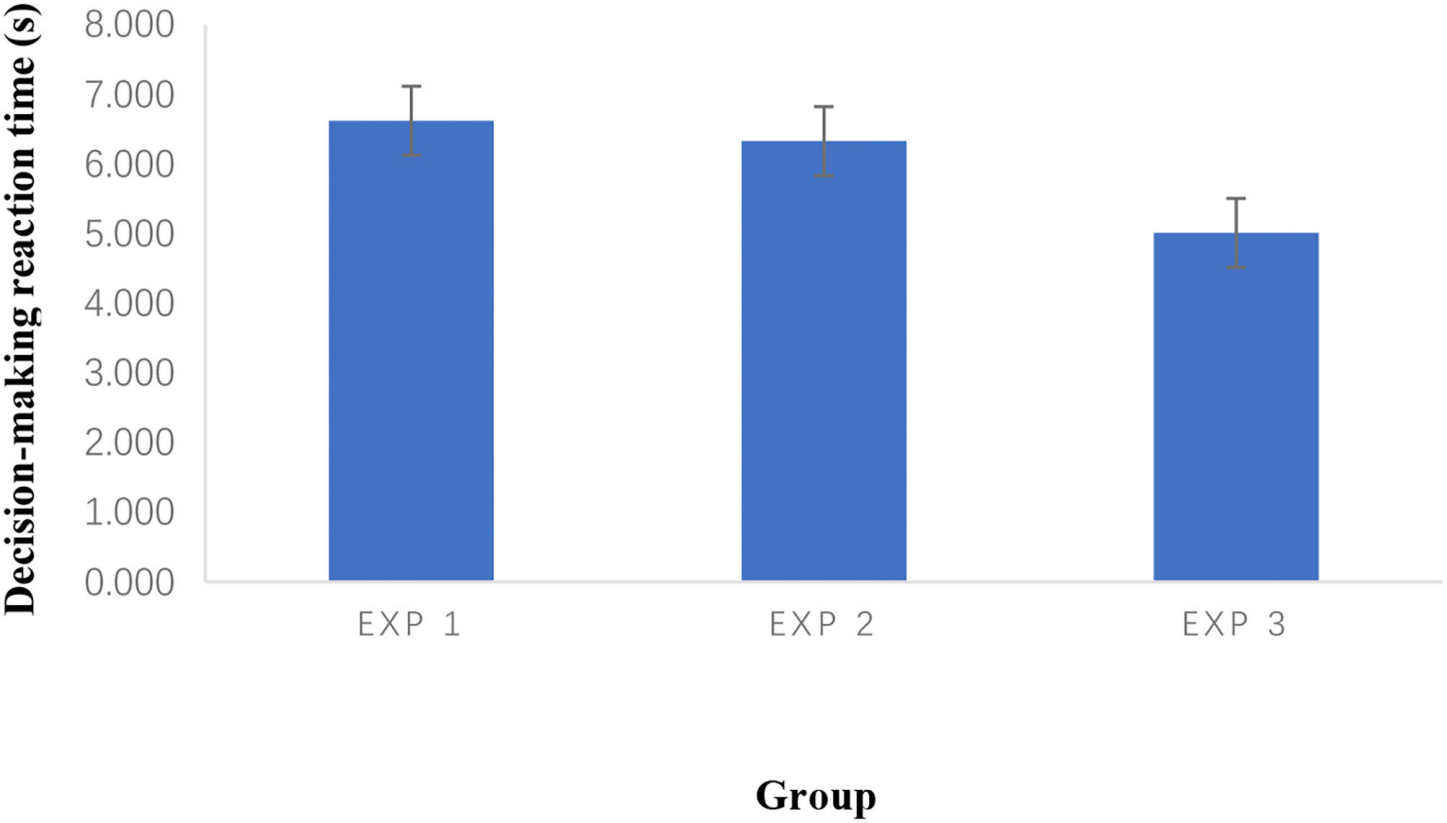
The mean of reaction time at post-test.

Finally, Figure 6 indicates that there is a decrease from Exp 1 to Exp 3. Overall, the decrease from Exp 2 to Exp 3 is sharper than Exp 1 to Exp 2.

## Discussion

In line with ART, it is not surprising that decision-making was significantly enhanced in Exp 3 from pretest to post-test, which was measured by accuracy and reaction time, for the directed attention was improved by nature exposure intervention, and players can focus more on relevant information in a decision-making task. However, Exp 2 only improved on their reaction time, and the accuracy did not increase significantly. The explanation can be found in a study by Rozand et al. (2015) on the impacts of mental fatigue for accuracy-speed ratios in taping actions with different difficulty indices. In the study, mental fatigue mainly influenced reaction time rather than accuracy. The important similarity of these two studies is that both emphasize speed and accuracy. Even though the subjects in these works were requested to conduct the tasks as accurately and quickly as possible, the tasks' limitations emphasized either accuracy or speed. The experiment of tapping tasks emphasized accuracy. If two subsequent targets are incorrect, the trial would be terminated by default. In this study, the timer in the trial would be awarded by subjects, indicating that the measurement of decision-making is time-based. Players may try to increase their speed, sacrificing accuracy when they are mentally fatigued during trials. Therefore, the finding proved that completing speed-accuracy trade-off existed when soccer players were mentally fatigued in making a decision. This finding suggested a more conservative strategy for mentally fatigued players. They may decide to pass the ball to a teammate under less defensive pressure rather than risking a pass to a marked teammate with a better attacking position.

The most exciting finding is in Exp 3, which significantly improves accuracy and reaction time. These improvements were not from lower RPE, for they were not significantly different in all groups at each test (Figure 2). Also, participants in Exp 3 cannot show improvements with any psychological technique, such as mindfulness, for doing this regulation requires participants' experience (Axelsen et al., 2020). The potential mechanism of impaired decision-making is recognized as the increase of adenosine and the corresponding decrease of dopamine in mentally fatigued players because mental fatigue tasks activated ACC (Smith et al., 2018; McMorris, 2020). The ACC plays a crucial role in attention allocation (Boksem et al., 2005). On the contrary, nature exposure may increase the dopamine in ACC (Darna et al., 2015). Thus, the counteractive effect of mental fatigue may appear in attention allocation, which enhanced directed attention and reduced stimulus-driven attention (Laumann et al., 2003; Berto, 2005).

Since soccer game is unpredictable in a continuously changing environment that requires the maintenance of alert states for a diverse set of indicators (Afonso et al., 2012), players must use directed attention, become less exposed to irrelevant distractors (e.g., cluster and stress), and become more focused on relevant indicators (e.g., teammates' movements and the ball's trajectory). By directing the attention to relevant indicators, players can use the perceived information, allowing the anticipating unfolding scenarios, leading to better decision-making in the task's constraints.

Another explanation for the improvement in Exp 3 is about executive function. Kaplan and Berman (2010) indicated that directed attention is an expected resource for self-regulation and executive function. In line with the strength model of self-regulation, the term resource implies that something can be finite and depleted by continuous demand. Directed attention is recognized as the resource and cross-domain convergence (Kaplan and Berman, 2010). When directed attention is increased significantly, self-regulation and executive function may also increase. Besides self-regulation, the executive function plays a crucial role in decision-making because soccer is an open-skill sport that requires players to continue making decisions quickly and accurately in a dynamically unpredictable environment. So, players must perceive and address information (e.g., working memory) to find the best solution in a constraint of time (e.g., planning, reasoning, and creativity) (Sakamoto et al., 2018). The nature exposure in Exp 3 (12.50 min) increased the resource for executive function; therefore, players performed better in their decision-making.

There were no significant differences in soccer decision-making among the three experimental groups. However, there was a trend of improving the decision-making reaction time of Exp 3 more than Exp 2 in Figure 6, exceptionally, when the accuracy was almost maintained in Exp 3 (Figure 5).

The primary reason to induce the difference in decision-making between Exp 2 and Exp 3 is the duration of the intervention. According to ART, directed attention can rest or restore when involuntary attention is attracted by nature scenes (Kaplan and Berman, 2010).

Thus, the duration of attracting involuntary attention is essential. In short, the duration of nature exposure can determine the subsequent improvement when the quality of nature scenes is fixed. This study argued that the shorter duration of nature exposure in Exp 1 (4.17 min) and Exp 2 (8.33 min) could not restore the directed attention as much as the longer duration (12.50 min) in Exp 3. In addition, each stimulus material used in Exp 3 staying duration is longer (30 s). The duration of nature exposure must be 12.50 min to improve the decision-making in mentally fatigued university soccer players. Therefore, soccer players could use nature stimuli to do visualization or guided imagery training. In this way, it could be a resource that players can use to improve mental fatigue in many scenarios (e.g., training or competition); in the same way, there is a resource and technique that can be used to work on the level of activation. Moreover, some common everyday pre-match activities could induce mental fatigue, such as traveling to away matches, education in courses, and team talks (Thompson et al., 2020). University soccer teams could apply the intervention before training or competition to improve mental fatigue level and players' performance.

## Conclusion

Consistent with ART, nature exposure significantly improved decision-making in mentally fatigue university players. However, it depends on the duration of the intervention when the nature scenes are fixed. Based on this study, the duration must be 12.50 min (each stimulus staying 30 s) to counteract mental fatigue and improve soccer decision-making, for the other two durations (4.17 and 8.33 min) were not enough to rest directed attention and induce restoration. Future studies may consider applying the intervention to other sports (e.g., basketball, table tennis, and cricket).

## Limitation

This study poses a few noteworthy limitations. First, the inducement of a prolonged computer version of the Stroop task. The cognitive demands of this task (e.g., sustained attention and inhibitory control) are present during soccer games; however, players usually do not perform this task before the games. Future studies may consider investigating mental fatigue from the actual pre-match activities.

Second, this study recruited the subjective measurement of mental fatigue; however, the objective measurement should be acknowledged; especially, the most recent studies demonstrated that the central nervous system-related mechanisms (e.g., cortical arousal, brain's direct current, and HRV) play an unparallel role on the development of fatigue (De La Vega et al., 2021b). However, since mental fatigue only remained elevated in a certain period (≥50 min) following the 45-min Stroop task (Smith et al., 2019), to maintain the validity and reliability of the experiment, the tool (VAS) with a minimum time requirement was used. Nevertheless, future studies may try to employ objective indicators to assess mental fatigue, such as HRV, for it is modified by cognitive and attentional activities in sports (e.g., chess) (Fuentes et al., 2018; Fuentes-García et al., 2019). Also, there are valid tools that can be used to monitor the psychological condition conveniently, such as fatigue evaluation app (De La Vega et al., 2021a) and online evaluation platform (Reigal et al., 2020).

Third, the virtual reality exposure technique is outstanding in full immersion with some devices (Diemer et al., 2015). However, the cost is a major consideration to apply the intervention practically among university soccer teams.

Finally, this study only recruited active control groups with urban content. Future studies may consider employing control groups without any content. That is, participants just simply rest or relax in certain durations.

## Data Availability Statement

The original contributions presented in the study are included in the article/supplementary material, further inquiries can be directed to the corresponding author/s.

## Ethics Statement

The studies involving human participants were reviewed and approved by Universiti Putra Malaysia Ethics Committee. The participants provided their written informed consent to participate in this study.

## Author Contributions

All authors participated in the design, documentation, development, and writing of the manuscript, reviewed the article, were responsible for its contents, and provided responsible for the final version. All authors contributed to the article and approved the submitted version.

## Conflict of Interest

The authors declare that the research was conducted in the absence of any commercial or financial relationships that could be construed as a potential conflict of interest.

## Publisher's Note

All claims expressed in this article are solely those of the authors and do not necessarily represent those of their affiliated organizations, or those of the publisher, the editors and the reviewers. Any product that may be evaluated in this article, or claim that may be made by its manufacturer, is not guaranteed or endorsed by the publisher.
